# Goal Directed Worry Rules Are Associated with Distinct Patterns of Amygdala Functional Connectivity and Vagal Modulation during Perseverative Cognition

**DOI:** 10.3389/fnhum.2016.00553

**Published:** 2016-11-02

**Authors:** Frances Meeten, Graham C. L. Davey, Elena Makovac, David R. Watson, Sarah N. Garfinkel, Hugo D. Critchley, Cristina Ottaviani

**Affiliations:** ^1^Institute of Psychology, Psychiatry and Neuroscience, King’s College LondonLondon, UK; ^2^Department of Psychiatry, Brighton and Sussex Medical School, University of SussexBrighton, UK; ^3^School of Psychology, University of SussexBrighton, UK; ^4^Neuroimaging Laboratory, Santa Lucia FoundationRome, Italy; ^5^Sackler Centre for Consciousness Science, University of SussexBrighton, UK; ^6^Sussex Partnership NHS Foundation Trust SussexSussex, UK

**Keywords:** generalized anxiety disorder, perseverative cognition, amygdala, functional connectivity, worry stop rules

## Abstract

Excessive and uncontrollable worry is a defining feature of Generalized Anxiety Disorder (GAD). An important endeavor in the treatment of pathological worry is to understand why some people are unable to stop worrying once they have started. Worry perseveration is associated with a tendency to deploy goal-directed worry rules (known as “as many as can” worry rules; AMA). These require attention to the goal of the worry task and continuation of worry until the aims of the “worry bout” are achieved. This study examined the association between the tendency to use AMA worry rules and neural and autonomic responses to a perseverative cognition induction. To differentiate processes underlying the AMA worry rule use from trait worry, we also examined the relationship between scores on the Penn State Worry Questionnaire (PSWQ) and neural and autonomic responses following the same induction. We used resting-state functional magnetic resonance brain imaging (fMRI) while measuring emotional bodily arousal from heart rate variability (where decreased HRV indicates stress-related parasympathetic withdrawal) in 19 patients with GAD and 21 control participants. Seed-based analyses were conducted to quantify brain changes in functional connectivity (FC) with the amygdala. The tendency to adopt an AMA worry rule was associated with validated measures of worry, anxiety, depression and rumination. AMA worry rule endorsement predicted a stronger decrease in HRV and was positively associated with increased connectivity between right amygdala and locus coeruleus (LC), a brainstem noradrenergic projection nucleus. Higher AMA scores were also associated with increased connectivity between amygdala and rostral superior frontal gyrus. Higher PSWQ scores amplified decreases in FC between right amygdala and subcallosal cortex, bilateral inferior frontal gyrus, middle frontal gyrus, and areas of parietal cortex. Our results identify neural mechanisms underlying the deployment of AMA worry rules. We propose that the relationship between AMA worry rules and increased connectivity between the amygdala and prefrontal cortex (PFC) represents attempts by high worriers to maintain arousal and distress levels in order to feel prepared for future threats. Furthermore, we suggest that neural mechanisms associated with the PSWQ represent effortful inhibitory control during worry. These findings provide unique information about the neurobiological processes that underpin worry perseveration.

## Introduction

Worry is a cognitive activity experienced by most individuals, but for some this activity can become pathological, uncontrollable and distressing, and lead to regular bouts of perseverative cognition that negatively affects many forms of daily functioning. Pathological worry of this kind is the cardinal diagnostic feature of Generalized Anxiety Disorder (GAD; DSM-5, American Psychiatric Association, 2013), and is also an important transdiagnostic process, which contributes to the symptoms observed in a range of other psychopathologies (Barlow et al., 2004; Ehring and Watkins, 2008).

A commonly cited approach to understanding worry has conceptualized it as a strategy of cognitive avoidance in response to threat (Borkovec, 1994; Borkovec et al., 2004). Individuals with GAD perceive the world as being a threatening place and one way of managing physiological and psychological feelings of fear is to anticipate what may happen in the future and try to prepare oneself for them. From this perspective, worry is considered as a mental attempt to solve problems (Sibrava and Borkovec, 2008). However, on a more mechanistic level, proximal models of individual pathological worry have only recently been developed (Hirsch and Mathews, 2012; Davey and Meeten, in press), and will be required to understand the individual neurological and psychological mechanisms that generate a worry experience that is perseverative, seemingly uncontrollable, and increasingly distressing as the bout continues.

There is a need to examine the psychological processes that underlie pathological worry, and differentiate it from non-clinical worry, in terms of autonomic and neurobiological correlates of worry. One approach is to explore the goal-directed rules that people use when worrying, to understand why some people persevere with worry after others have stopped. For most people, worrying has a purpose, whether it be to solve perceived problems of daily living (Davey, 1994), as an attempt to repair negative mood (Schwarz and Clore, 1983), or as a means to try and ensure that “bad” things don’t happen or to avoid future catastrophes (Davey et al., 1996; Breitholtz et al., 1998; Borkovec et al., 1999; Wells, 2010). Worrying of this kind usually comes with a set of implicit goal-directed rules that are deployed to maximize goal attainment (Chaiken et al., 1989; Martin et al., 1993). These rules don’t necessarily tell the worrier how to achieve the goal, but they have a motivational influence by stressing the importance of the goal and activating processes for monitoring whether the goal has been achieved (Davey, 2006; Davey and Meeten, in press).

The endorsement of goal-directed worry rules is highly correlated with a variety of worry-relevant variables (Davey et al., 2005), including measures of trait worry (as measured by the Penn State Worry Questionnaire, PSWQ; Molina and Borkovec, 1994) and beliefs about the positive consequences of worry (as measured by the Consequences of Worry Scale; Davey et al., 1996). Furthermore, the reported use of goal-directed rules significantly predicts perseveration on behavioral measures of catastrophic worrying (Davey et al., 2005).

A recent cognitive model of the perseverative worry bout (Davey and Meeten, in press) describes how potential worries or threats activate the pathological worrier’s positive beliefs about a need to worry (Wells, 2007, 2010), and how these beliefs are operationalized in the deployment of goal-directed rules for worrying. Consequently, the pathological worrier “continues to worry until he/she assesses that he/she will be able to effectively cope with anticipated threat” (Wells, 2007, p.19). Identified threats act to prime habitual goal-directed worry rules in an automatic fashion (Bargh, 1989; Aarts and Dijksterhuis, 2000), and these same strict rules for completion of the worry bout also directly contribute to perseveration (ensuring all eventualities are considered).

In addition to psychological models of pathological worry, there have been recent attempts to examine the neurological and autonomic correlates of worry. Research has highlighted specific autonomic, and neurobiological responses to worry, and perseverative cognition including a number of somatic reactions (Brosschot et al., 2006; Ottaviani et al., 2016a), one of which is reduced heart rate variability (HRV). Reduction in HRV is a recognized feature of worry and has been shown to mirror cognitive and emotional inflexibility in worry and rumination (Ottaviani et al., 2013, 2015). Borkovec and Hu (1990) demonstrated that worry can reduce emotional responding to a negative stressor (where worry is assumed to function as a way of avoiding negative emotional states). Reduced HRV can be seen as the physiological component of cognitive perseveration (Thayer et al., 1996).

Brain imaging studies have shown reduced connectivity between the amygdala and prefrontal cortex (PFC) in GAD patients as compared to healthy controls (HC; Monk et al., 2008; Roy et al., 2013). This pattern of aberrant connectivity between amygdala and pre-frontal areas is associated with poor emotional regulation (Borkovec et al., 2004; Banks et al., 2007; Hamm et al., 2014).

The relationship between autonomic and neurobiological signatures of pathological worry has recently been examined by Makovac et al. (2016) where resting state functional magnetic resonance imaging (fMRI) techniques and physiological recordings were combined to characterize the interplay between psychological and physiological symptoms of worry. This study reinforced earlier findings of lower connectivity between the right amygdala and pre-frontal areas (namely right superior frontal gyrus, right paracingulate/anterior cingulate cortex (ACC) and right supramarginal gyrus) in individuals with GAD. The study also highlighted shared neural correlates (centered on amygdala connectivity) of worry and autonomic dysregulation in GAD participants, which suggests a common mechanism underlying affective and physiological symptomatology. Aberrant connectivity between the amygdala and PFC is an established resting state finding in high anxious populations, however the functional differences between anxious and non-anxious populations while engaging in a worry bout are less well understood.

The purpose of the present study is to supplement this knowledge of the role of the deployment of goal-directed rules (i.e., “as many as can” (AMA) stop rules) in pathological worrying. Functional changes in brain activity and autonomic bodily responses (HRV) were measured following a perseverative cognition induction and related to the use of goal-directed worry stop rules. Further, we also report the relationship between neural and autonomic responses following the perseverative cognition induction and individual differences in the level of trait worry (measured using the PSWQ; Meyer et al., 1990). Together these approaches aimed to enhance our understanding of pathological worrying by characterizing associations between stop-rule deployment and functional brain activity and autonomic arousal state.

It was hypothesized that:

1. AMA stop rules correlate with validated measures of worry, anxiety and rumination.
2. Based on the finding that a perseverative cognition induction resulted in increased connectivity between the amygdala and PFC in GAD participants (Makovac et al., 2016), we predicted that trait AMA stop-rule adoption is associated with a shift towards increased connectivity between the amygdala and PFC following the perseverative cognition induction.

## Materials and Methods

### Participants

One participant who did not complete the full experiment was excluded and the final sample encompassed 19 patients (17 women, 2 men; mean age = 29.58 (6.93) years) who met diagnostic criteria for GAD and 21 HC (18 women, 3 men; mean age = 28.67 (9.45) years). Only one participant was non-Caucasian. Patients and HC were recruited from public advertisement. All participants were right-handed, native English speakers, and had normal or corrected vision. Exclusion criteria were: age below 18 years, past head injury or neurological disorders, history of major medical or psychiatric disorder (other than GAD and co-morbid depression in the patients), cognitive impairment, history of substance or alcohol abuse or dependance, heart disease, obesity (body mass index >30 kg/m^2^), pregnancy, claustrophobia or other MRI exclusions. Two GAD patients were included who took long-term medication (1 Citalopram, 1 Pregabalin) at the time of the study. All other participants were medication free. All participants provided written informed consent. The study was approved by the National Research Ethics Service (NRES) with local approval from the Brighton and Sussex Medical School Research Governance and Ethics Committee. Participants were compensated for their time.

### Procedure

The Structured Clinical Interview for DSM-IV (SCID) was administered by a trained postdoctoral fellow (FM) to patients and controls to confirm/exclude the diagnosis of GAD. Participants then completed socio­demographic and dispositional traits questionnaires. Participants were subsequently familiarized with the neuroimaging environment, connected to the physiological recording equipment, and then underwent the MRI protocol.

### Questionnaires

#### Worry Stop Rule Checklist

The Worry Stop Rule Checklist (Davey et al., 2005) is a measure designed to assess trait stop rules, or specific beliefs used to decide when to discontinue a worry bout or episode (Davey et al., 2005). The measure consists of two subscales. The first scale consists of 10 items measuring the degree to which individuals endorse goal-directed or AMA stop rules while worrying (e.g., “I must keep worrying about this, otherwise things won’t get done properly”). The second scale consists of 9-items that assess the degree to which individuals use a “feel like continuing” (FL) stop rule (e.g., “Stop worrying- in the long run this just won’t matter very much”). This measure has shown to have adequate internal consistency (*α* = 0.82–0.88), and validity, as moderate to strong correlations have been found between this measure and the PSWQ (Davey et al., 2005, 2007; Turner and Wislon, 2010).

#### Penn State Worry Questionnaire

The PSWQ, (Meyer et al., 1990) is the most widely used valid measure of the frequency and intensity of worry. The PSWQ consists of 16-items (e.g., “Many situations make me worry”), which are rated on a 5-point Likert scale ranging from (1) “not at all typical of me” to (5) “very typical of me”. The PSWQ has good test-retest reliability (*r* = 74–0.93; Molina and Borkovec, 1994), internal consistency (*α* = 90; Brown et al., 1992), and discriminant validity (Meyer et al., 1990).

#### State-Trait Anxiety Inventory Form Y

The State-Trait Anxiety Inventory Form Y (STAI; Spielberger et al., 1983) consists of two parts, each comprising 20 questions. STAI-Y1 measures state anxiety, that is the respondent’s current level of anxiety, by asking how they feel “right now”, with a four-point scale of responses from “not at all” to “very much so” for statements such as “I am tense” and “I feel nervous”. STAI-Y2 measured trait anxiety, or differences in proneness to anxiety, by asking how participants generally feel, with a four-point scale from “almost never” to “almost always” for statements such as “I feel like a failure” and “I have disturbing thoughts”. Internal consistency coefficients for the scale have ranged from 0.86 to 0.95; test-retest reliability coefficients have ranged from 0.65 to 0.75 over a 2-month interval (Spielberger et al., 1983).

#### Ruminative Responses Scale

The Ruminative Response Scale (RRS) is a subscale of the Response Styles Questionnaire (Nolen-Hoeksema and Morrow, 1991) consisting of 24 items, revealed as highly reliable and valid in measuring reactions to experiencing negative emotions (*α* = 0.92). Individuals must respond to a series of captions such as “Think about how passive and unmotivated you feel” where possible responses are “almost never/sometimes/often/almost always”.

### Experimental Design

During scanning, participants underwent a series of four 5-min resting state periods, each followed by a 6-min easy visuomotor tracking task (described elsewhere; Ottaviani et al., 2016b). During resting state periods participants were instructed to rest with their eyes open without thinking of anything and not falling asleep. After the second or third resting block, participants underwent a recorded verbal induction procedure designed to engender perseverative cognition:

“Next I would like you to recall an episode that happened in the past year that made you feel sad, anxious, or stressed or something that may happen in the future that worries you. Then, I would like you to think about this episode in detail, for example about its possible causes, consequences, and your feelings about it. Please keep thinking about this until the end of the next tracking task. Thank you. Please take as much time as you need to recall the episode and press the button whenever you are ready”.

To assess state levels of perseverative cognition, at the end of each resting-state period, participants rated their thoughts over the preceding period.

The perseverative cognition induction has established efficacy in eliciting worrisome thoughts that were reproduced in this group (described Makovac et al., 2016).

### Physiological Data Processing

Cardiac signal was collected using MRI-compatible finger pulse oximetry (8600FO; Nonin Medical) recorded digitally (via a CED power 1401, using Spike2 v7 software; Cambridge Electronic., Design CED). Pulse data were manually checked and corrected for artifacts. After extracting inter-beat intervals, HRV was estimated by calculating the root mean square successive difference (RMSSD) a reliable parameter for assessing vagally-mediated HRV (Task Force of the European Society of Cardiology and the North American Society of Pacing and Electrophysiology, 1996). RMSSD was derived using RHRV 4.0 analysis software^1^. Individual HRV estimates were obtained for the duration of each resting state scanning period. Attention was given to HRV measures before (Pre) and after (Post) the perseverative cognition induction.

### MRI Acquisition and Preprocessing

MRI images were acquired on a 1.5-Tesla Siemens Magnetom Avanto scanner. Structural volumes were obtained using the high-resolution three-dimension magnetization-prepared rapid gradient-echo sequence (HiRes3DMPRAGE). Functional datasets used T2*weighted echoplanar imaging (EPI) sensitive to blood oxygenation level dependent (BOLD) signal (TR = 2.52 s, TE = 43 ms, flip-angle (FA) = 90° 34 slices, slice thickness = 3 mm; FOV = 192 mm, voxel size 3 mm × 3 mm × 3 mm).

Data were pre-processed using Statistical Parametric Mapping (Wellcome Department of Imaging Neuroscience; SPM8^2^), and in-house software implemented in Matlab (The Mathworks Inc, Natick, MA, USA). For each participant, the first four volumes of the fMRI series were discarded to allow for T1 equilibration effects. The pre-processing steps included correction for head motion, compensation for slice-dependent time shifts, normalization to the EPI template in standard space (MNI) coordinates provided with SPM8, and smoothing with a3D Gaussian Kernel with 8 mm^3^ full-width at half maximum. The global temporal drift was removed using a 3rd order polynomial fit. To remove other potential sources of bias, data was further filtered regressing against the realignment parameters, and the signal averaged over whole brain voxels. Then, all images were filtered by a phase-insensitive band-pass filter (pass band 0.01–0.08 Hz) to reduce the effect of low frequency drift and high frequency physiological noise.

### Statistical Analyses

#### Questionnaire, Behavioral, and HRV Analyses

All data are expressed as means (±SD). Differences at *p* ≤ 0.05 are regarded as significant unless corrections for multiple comparisons are stated. Data analysis was performed with SPSS 22.0 for Windows (SPSS Inc., Chicago, IL, USA). Pearson’s *r* correlations were conducted to test for associations between worry stop-rules (AMA and FL) and validated questionnaire measures of worry, anxiety, depression and rumination.

### Seed-Based fMRI Analysis

Given the organization of the brain into functional networks, functional connectivity (FC) is a valuable tool as it measures inter-regional synchrony of low frequency fluctuations in BOLD fMRI (Biswal et al., 1995; Fox et al., 2005). In the present study, we used seed-based analyses as our approach was hypothesis-driven.

Anatomical ROIs were constructed using an anatomical toolbox in SPM (Tzourio-Mazoyer et al., 2002) for bilateral amygdala. The average resting state fMRI time-series over the ROIs were extracted for GAD and HC groups. These time series were then used as a regressor in a 1st level SPM analysis.

A correlational analysis was then carried out between the difference in FC after the perseverative cognition induction (Δ = post − pre-induction) and AMA, FL and PSWQ scores, using a *t*-test with Group (GAD, HC) as main factor and AMA, FL and PSWQ scores as variables of interest.

## Results

There were no significant group differences for any of the assessed socio-demographic and lifestyle variables (see Makovac et al., 2016).

### Questionnaires

The GAD group had significantly higher scores on the Worry Stop Rule Checklist “AMA” scale [GAD; *M* = 36.28, *SD* = 5.62, HC; *M* = 22.57, *SD* = 5.42, *t*_(38)_ = 7.35, *p* = < 0.001, *r* = 0.77] and significantly lower scores on the “FL” scale, as compared to the HC group [GAD; *M* = 18.56, *SD* = 6.24, HC; *M* = 29.10, *SD* = 9.21, *t*_(38)_ = 4.19, *p* = < 0.001, *r* = 0.56]. The GAD group also had significantly higher scores on the PSWQ, the STAI, and the RRS and significantly lower HRV than the HC group (see Makovac et al., 2016).

Correlation analysis was performed to examine the relationship between the Worry Stop Rule Checklist and validated trait measures of worry, rumination and anxiety (see Table 1). An accepted significance level *p* = 0.01 was used in correction for multiple comparisons. The AMA stop rule scale was significantly positively correlated with the PSWQ, the STAI-Y2 and RRS and the FL scale was significantly negatively correlated with the PSWQ and the STAI-Y2 measures, and shows a trend to negative correlation with RRS.

**Table 1:** **Pearson’s *r* correlations between worry rules as measured by the Stop Rule Checklist and trait measures of worry (Penn State Worry Questionnaire, PSWQ), anxiety (State-Trait Anxiety Inventory Form, STAI), and rumination (Ruminative Response Scale, RRS)**.

Questionnaires	AMA stop rule	FL stop rule	PSWQ	STAI-Y2	RRS
AMA stop rule	–	−0.626**	0.686**	0.703**	0.491**
FL stop rule	−0.626**	–	−0.633**	−0.595**	−0.328*

Note: *p < 0.05, **p ≤ 0.001.

### Heart Rate Variability (HRV)

HRV showed a significant reduction following the perseverative cognition induction. Change scores (post − pre) were negatively correlated with AMA stop rules, *r* = −0.45, *p* = 0.004 and positively correlated with FL stop rules, *r* = 0.40, *p* = 0.01. There was no significant relationship between PSWQ and pre to post induction change in HRV, *r* = −0.27, *p* = 0.09.

### Correlation Between Pre- to Post-Induction Changes in Amygdala Functional Connectivity and Scores on the Worry Stop Rule Checklist

**Figure 1:**
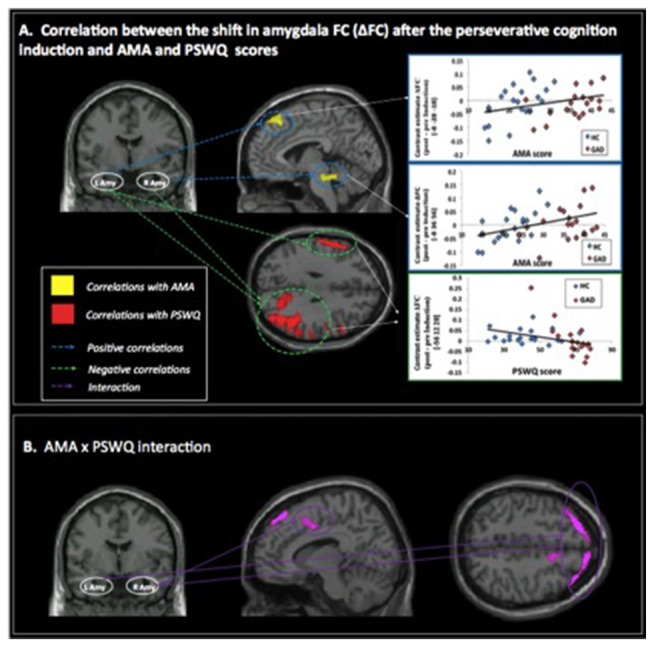
**(A)** Correlations between pre- to post-induction changes [Δ = post − pre-induction] in amygdala functional connectivity (FC) and “as many as can” (AMA) and trait worry (Penn State Worry Questionnaire [PSWQ]) scores. Positive correlations are represented with blue dashed lines and indicate that higher AMA scores were associated with stronger FC increases following the perseverative cognition induction; negative correlations are represented with a green dashed line and indicate that higher scores on the PSWQ were associated with stronger decreases in amygdala FC following the perseverative cognition induction. **(B)** Significant AMA × PSWQ interactions when scores on both questionnaires were simultaneously entered in the model.

A positive correlation was obtained between AMA score and pre- to post-induction changes in FC [Δ = post − pre-induction] between right amygdala and locus coeruleus (LC), a brainstem (pons) center for noradrenergic projections up and down the neuraxis (Table 2). Similarly, a positive correlation was observed between AMA score and Δ FC between left amygdala and superior frontal gyrus. Thus, higher AMA scores predicted a stronger increase in FC (i.e., higher Δ FC values) between amygdala and LC and superior frontal gyrus, respectively (see Figure 1).

**Table 2:** **Correlations between pre- to post-induction changes in amygdala functional connectivity (FC) and scores on the “as many as can” (AMA) subscale of the Stop Rule Checklist and PSWQ and significant interaction effects when both measures are simultaneously entered into the model**.

Brain region		Cluster		Voxel			
	Side	*k*	*p FWE*	*Z*	*MNI xyz*		
**Positive correlation with AMA**
*Right amygdala seed*
Locus coeruleus	R	374	0.004	4.31	−8	−28	−18
*Left amygdala seed*
Superior frontal gyrus	L	983	0.000	4.32	−8	36	56
	R			4.02	8	38	56
Middle frontal gyrus	R			3.69	36	34	48
**Negative correlation with PSWQ**
*Right amygdala seed*
Lateral occipital cortex	R	1311	0.000	5.16	40	−78	26
Precuneus	R			4.53	20	−64	32
Precentral gyrus	R	608	0.000	4.56	56	−2	22
	L	559	0.000	4.35	−56	6	38
Middle frontal gyrus	R	353	0.000	3.79	34	−2	46
*Left amygdala seed*
Inferior frontal gyrus	L	820	0.000	5.23	−56	12	26
Lateral occipital cortex	R	859	0.000	4.19	40	−78	24
Angular gyrus	R			4.00	44	−50	32
**AMA × PSWQ interaction**
*Right amygdala seed*
Superior frontal gyrus	R	525	0.000	4.42	10	40	56
Middle frontal gyrus	L			3.53	−32	30	50
	R			3.18	38	32	48
Anterior cingulate cortex	R	237	0.051	3.98	16	−4	40
*Left amygdala seed*
Superior frontal gyrus	R	751	0.000	4.33	10	42	56
	L			4.03	−22	40	50
Middle frontal gyrus	R			3.76	36	36	48

### Correlation Between Pre- to Post-Induction Changes in Amygdala Functional Connectivity and Scores on the Penn State Worry Questionnaire

A negative correlation was obtained between scores on the PSWQ and the change in FC between right amygdala and lateral occipital cortex, precentral gyrus and middle frontal gyrus and between PSWQ score and the change in FC between left amygdala and lateral occipital cortex and inferior frontal gyrus (for a complete list of brain areas, see Table 2). Higher PSWQ scores predicted a stronger decrease in amygdala FC with these areas from pre- to post-induction (see Figure 1).

### AMA × PSWQ Interaction

When both AMA and PSWQ were simultaneously entered in the regression model, the above mentioned amygdala connectivity patterns did not change. Moreover, a significant AMA × PSWQ interaction emerged for the FC between the bilateral amygdala and areas of the middle frontal gyrus, bilaterally. A significant AMA × PSWQ interaction also emerged for the FC between the right amygdala and the ACC. In detail, higher scores on the PSWQ were associated with decreased amygdala FC with these areas, whereas higher scores on AMA were associated with increased amygdala connectivity following the perseverative cognition induction (see Table 2 and Figure 1).

## Discussion

A recent model conceptualizes AMA goal-directed worry stop rules within an integrated network of behavioral and cognitive responses that occur during perseverative worry (Davey and Meeten, in press). A key aim of the present article was to extend our knowledge of perseverative worry more generally, and goal directed worry rules specifically, by examining the neurobiological processes that are related to AMA stop rule use and compare this with processes associated with a measure of trait worry (PSWQ) after a perseverative cognition induction. We examined responses to the Worry Stop Rule Checklist (Davey et al., 2005) by individuals with GAD as compared to the HC group. The GAD group scored significantly higher on the AMA subscale and significantly lower on the FL subscale than the HC group. The AMA subscale was also significantly positively correlated with validated measures of trait worry, anxiety and rumination. To our knowledge, this is the first time that the Worry Stop Rule Checklist has been completed by individuals with GAD. The present findings are consistent with previous research, which has shown that in a non-clinical sample, the tendency to adopt an AMA approach to worrying was positively associated with higher PSWQ scores and beliefs about positive and negative consequences of worry (Davey et al., 2005). Furthermore, previous research has shown that, over the course of a catastrophic worry task, individuals have a tendency to shift from endorsing AMA stop rules at the outset of the task, to FL rules at the end of the task (Davey et al., 2007). This suggests that FL stop rules are associated with cessation of worry.

Using seed-based fMRI analysis, we found that GAD participants with high AMA worry rule endorsement displayed larger decreases in HRV after a perseverative cognition induction and also displayed increased connectivity between right amygdala and brainstem. Higher AMA scores were also coupled to increased connectivity between amygdala and rostral superior frontal gyrus. In contrast, higher PSWQ scores were associated with stronger post induction decreases in FC between right amygdala and more ventral and lateral frontal regions (subcallosal cortex, bilateral inferior frontal gyrus).

The AMA worry rules and PSWQ shared a representation within the middle frontal gyrus, a region implicated as part of the cognitive regulation network (e.g., effortful regulation of affect), where activity is typically inversely correlated with spontaneous activity in the amygdala (Roy et al., 2009). Interestingly, we observed that lower scores on the PSWQ were associated with increased amygdala FC with this area during the perseverative cognition induction (i.e., a negative correlation), whereas higher scores for AMA were associated with increased amygdala connectivity (i.e., a positive correlation). Previous research has shown that increased connectivity between the right amygdala and left middle frontal gyrus is observed during threatening scenarios (Gold et al., 2015). Similarly, in the induction of worry in elderly patients with GAD, participants displayed enhanced connectivity between the paraventricular nucleus seed and middle frontal gyrus (Andreescu et al., 2015). Moreover, decreased amygdala FC with the middle frontal gyrus is observed after emotion regulation training (Li et al., 2016). Thus our findings enhance a growing literature concerning the contribution of middle frontal gyrus to affective regulation.

In the present study, we report that as an individual’s worry score (PSWQ) decreased, the perseverative cognition induction produced a greater increase in FC between the amygdala and the middle frontal gyrus. Makovac et al. (2016) reported that a HC group consistently displayed greater connectivity between the right amygdala and frontal pole regions (right superior frontal gyrus) compared to GAD patients. One possibility is that the present findings reflect attempts at effective top-down control of the amygdala where low anxious individuals may curb a worry bout through emotion regulation strategies such as reappraisal (e.g., Ochsner and Gross, 2005). Furthermore, connectivity between the amygdala and PFC has predicted lower levels of anxiety and effective emotional regulation (Kim et al., 2011).

In the present study, findings concerning trait worry (as examined by the PSWQ) support previous research findings in a GAD population (Hilbert et al., 2014). However, we propose that the AMA stop rule endorsement captures a different aspect of worry, which relates to the perseverative nature of pathological worry. For example, high anxious individuals have been shown to consider worry as a useful strategy to cope with threat and catastrophic worriers endorse positive beliefs about worry (Wells, 2004). We propose that the association between AMA stop rule endorsement and increased connectivity between the amygdala and middle frontal gyrus may capture successful attempts by the worrier to maintain chronic arousal and feelings of distress. For example, the contrast avoidance model of worry proposes that worry is reinforced because pathological worriers prefer to feel chronically distressed in order to prepare for the worst outcome (Newman and Llera, 2011). In high anxious individuals, a chronic state of cognitive and physiological readiness to deal with threat (e.g., worry perseveration) means that they avoid a potential future shift from a positive or benign mental state to a negative one (Newman and Llera, 2011).

Higher PSWQ scores were also uniquely associated with diminished pre- to post-induction connectivity of amygdala with the inferior frontal gyrus and the occipital cortex. Aberrant FC between these areas is implicated in functional impairments in socioemotional learning, anxiety, and self-referential insight (e.g., Singh et al., 2015). For example, exaggerated negative connectivity with lateral occipital cortex occurs in patients with social anxiety disorder (Pannekoek et al., 2013). The inferior frontal gyrus is also reported to show increased connectivity with the right amygdala during anxiety regulation engaged during threat exposure (Gold et al., 2015), consistent with a role in inhibitory control to cope with elevated task demands. Similarly, activation of lateral PFC with simultaneous attenuation of amygdala activity is reported during cognitive control of anxiety states from threat-related distractors and reappraisal of threat stimuli (for a meta-analysis see Buhle et al., 2014).

The pattern of results reported is consistent with a model of pathological worrying in which chronic worriers attempt to inhibit representations of the potential bad outcomes associated with the worry, while simultaneously maintaining arousal in order to seek out potential solutions to the issues raised by the worry. Thus, as a measure of the pathological frequency of worry, the PSWQ is not only associated with the deployment of AMA worry rules (to facilitate the finding of solutions to the worry through an internal narrative process), but also with attempts to inhibit threatening images of the potential worry entering conscious awareness. This latter process is consistent with the avoidance model of worry proposed by Borkovec et al. (2004) in which worry reflects a process of effortful inhibitory control of the fearful images associated with the worry, and this effect is implied by the relationship between PSWQ scores and diminished pre- to post-induction connectivity of amygdala with the inferior frontal gyrus and the occipital cortex. In contrast, the deployment of AMA worry rules is associated with increased connectivity between the right amygdala and the LC, which is the major noradrenergic nucleus of the brain and plays a central role in the regulation of arousal and autonomic activity. This finding supports the view that the deployment of AMA worry rules operationalizes a strategy to remain in a state of arousal reflecting preparedness for future negative outcomes and the need to persevere with worry in order to seek solutions for the worry. This is consistent with the contrast-avoidance model of worry that proposes that worry is reinforced because pathological worriers prefer to feel chronically distressed in order to prepare for the worst outcome. This also means that they avoid a potential future shift from a positive or benign mental state to a negative one (Newman and Llera, 2011). In addition, this interpretation is supported by the relevant physiological data showing that HRV exhibited significant reduction following the perseverative cognition induction, and HRV change scores were negatively correlated with AMA worry rule scores.

When both AMA and PSWQ are simultaneously entered in the model, the above-examined inverse amygdala connectivity patterns do not change, with the exception of a further relation between the right amygdala and the ACC that was positive for AMA and negative for PSWQ. The ACC is involved in the regulation of negative affect via its connections to the amygdala and the outflow to the autonomic system (reviewed in Bush et al., 2000; Lavin et al., 2013). A negative covariation between the amygdala and ACC in fear perception reflects reduced amygdala responses with greater ACC activity that is efficient top-down modulation of the amygdala (e.g., Das et al., 2005). Again, results are in agreement with scores on the PWSQ mirroring inhibitory control of fear and scores on AMA stop rules reflecting the proactive maintenance of fear, as indicated by greater ACC activity accompanied by enhanced amygdala activation.

In conclusion, we report that endorsement of AMA goal directed worry rules is associated with neural and autonomic responses to a perseverative cognition induction which can be characterized as an attempt to maintain a state of cognitive and physiological readiness for potential future feared outcomes. In contrast the PSWQ captures attempts to inhibit or avoid intrusive negative thoughts (Borkovec and Roemer, 1995). This is, to our knowledge, the first time that goal directed worry rules have been explored at a neurological level. Gaining a greater understanding about the psychological and physiological processes that drive worry perseveration will enable the development of more specific treatment approaches, which focus on factors that are likely to drive the excessive perseveration of worry that is reported in common psychopathologies such as GAD.

## Author Contributions

CO, HDC, FM, GCLD, DRW and SNG contributed to study design. CO, EM, SNG, DRW, HDC and FM contributed to data analysis. FM, GCLD, EM, CO, HDC, DRW contributed to writing the article.

## Conflict of Interest Statement

The authors declare that the research was conducted in the absence of any commercial or financial relationships that could be construed as a potential conflict of interest.

## Footnotes

^1^http://rhrv.r-forge.r-project.org/

^2^http://www.fil.ion.ucl.ac.uk/spm/
